# Measurement of Dimensions of Self-awareness of Memory Function and Their Association With Clinical Progression in Cognitively Normal Older Adults

**DOI:** 10.1001/jamanetworkopen.2023.9964

**Published:** 2023-04-25

**Authors:** Kayden J. Mimmack, Geoffroy P. Gagliardi, Gad A. Marshall, Patrizia Vannini

**Affiliations:** 1 Department of Neurology, Massachusetts General Hospital, Harvard Medical School, Boston; 2 Department of Neurology, Brigham and Women’s Hospital, Harvard Medical School, Boston, Massachusetts; 3 Center for Alzheimer Research and Treatment, Boston, Massachusetts

## Abstract

**Importance:**

The ability to separately explore 2 dimensions of self-awareness of memory function—increased and decreased awareness—in cognitively normal older adults provides an important opportunity to understand subtle changes in either direction in relation to risk of Alzheimer disease.

**Objective:**

To investigate the association of a novel measure for self-awareness of memory function with future clinical progression in individuals who were cognitively normal at baseline.

**Design, Setting, and Participants:**

This cohort study used data from the Alzheimer’s Disease Neuroimaging Initiative, a multicenter study. Participants were older adults who were cognitively normal (ie, Clinical Dementia Rating [CDR] global score of 0) at baseline and had at least 2 years of follow-up. Data were collected from June 2010 to December 2021 and pulled from the University of Southern California Laboratory of Neuro Imaging database on January 18, 2022. Clinical progression was defined as the first instance of 2 consecutive follow-up CDR scale global scores of 0.5 or greater.

**Main Outcomes and Measures:**

A traditional awareness score was measured by calculating the mean discrepancy between the participant and their study partner’s scores on the Everyday Cognition questionnaire. An unawareness or heightened awareness subscore was generated by capping item-level positive or negative differences at zero before averaging. The main outcome—risk of future clinical progression—was analyzed for each baseline awareness measure using Cox regression analysis. Longitudinal trajectories of each measure were additionally compared using linear mixed-effects models.

**Results:**

The 436-person sample included 232 (53.2%) female participants, with a mean (SD) age of 74.5 (6.7) years; 25 participants (5.7%) were Black, 14 (3.2%) Hispanic, and 398 (91.3%) White; and 91 participants (20.9%) clinically progressed over their period of observation. Survival analyses showed that a 1-point improvement on the unawareness subscore was associated with an 84% reduction in progression hazard (hazard ratio, 0.16 [95% CI, 0.07-0.35]; *P* < .001), or equivalently, a 1-point decrease was associated with a 540% increase in progression hazard (95% CI, 183% to 1347%), with no significant results for the heightened awareness or traditional scores.

**Conclusions and Relevance:**

In this cohort study of 436 cognitively normal older adults, unawareness, rather than heightened awareness, of memory decline was strongly associated with future clinical progression, providing further support that discordant self- and informant-reported cognitive decline may provide important information to practitioners.

## Key Points

**Question:**

Are separate, item-based measures of unawareness or heightened awareness of memory function associated with future progression from preclinical to symptomatic Alzheimer disease?

**Findings:**

In this cohort study of 436 older adults, worse unawareness scores were associated with a significantly higher hazard of clinical progression, with no significant results found for the heightened awareness or traditional awareness measures.

**Meaning:**

In this study, unawareness, rather than heightened awareness, was strongly associated with future clinical progression; isolating this dimension could provide greater sensitivity and specificity in investigation of awareness in Alzheimer disease.

## Teaser

This cohort study investigates the association of a novel measure for self-awareness of memory function with future clinical progression toward Alzheimer disease in individuals who were cognitively normal at baseline.

## Introduction

Loss of memory is a defining symptom of Alzheimer disease (AD),^1,2,3,4^ but not all patients are aware of their own cognitive decline. Such patients are said to display a lack of awareness, or *anosognosia*. Although anosognosia is highly prevalent at the AD dementia stage,^5^ previous longitudinal studies have shown that it may be present up to 2 to 3 years before an AD dementia diagnosis is made^6,7,8^ and is related to AD biomarkers.^6,9,10,11,12,13,14^ Its converse, heightened awareness, has also been shown to be present in the early stages of AD.^6,7^ Some individuals on the AD trajectory possess subjective insight into their early changes in cognition, outpacing their performance on objective measures and the external assessments of their friends, family members, and medical practitioners. It has been proposed that awareness may vary along a bidirectional continuum^15^ throughout the AD trajectory, often beginning with a period of heightened awareness before devolving into unawareness.^16^ However, previous studies have found that subjective concerns in the context of heightened awareness^6,7,17^ are common and not specific to AD, as concerns regarding memory changes may be due to other reasons, such as normal age-related changes, depression, anxiety, and nosophobia (ie, fear of disease).

Awareness is typically assessed in 1 of 3 ways^5,18^: (1) an interview with a clinician, (2) comparing the individual’s self-assessment on a memory questionnaire to objective cognitive scores, or (3) comparing the individual’s self-assessment to an external assessment by their study partner on the same memory questionnaire. The self-vs–study partner approach is traditionally scored by taking the difference of the respondents’ means on the questionnaire.^19^ Greater mean concerns by the study partner suggest loss of awareness in the participant, whereas greater mean concerns by the participant may indicate heightened awareness. However, the traditional scoring method has a major disadvantage: when individuals display unawareness in certain domains and heightened awareness in others, those differences are averaged out, making this measure insensitive to subtle changes in either direction. For example, an individual would be scored as aware in 2 discrepant situations: (1) when both the participant and their study partner are in agreement across all domains vs (2) when the participant is more sensitive to certain changes and less sensitive to others. While these 2 situations are treated as equivalent under the traditional approach, from a clinical point of view they are distinct, and each may provide important information to the practitioner. To attain sensitive measures of heightened awareness and unawareness, a scoring approach that isolates these 2 dimensions is needed.

In this study, we developed a new scoring approach for awareness questionnaires that separates the 2 dimensions of awareness—heightened awareness and unawareness—into 2 distinct subscores. First, we aimed to compare each of these new subscores with each other and with the traditional awareness score, examining their evolution over time. Our second aim was to examine the association of each of the 3 measures at baseline with future clinical progression from cognitively normal (CN) to mild cognitive impairment (MCI) or AD dementia. Based on previous findings,^6^ we hypothesized that greater baseline unawareness would be associated with impending clinical progression, and we expected this association to be weaker for heightened awareness.

## Methods

### Participants

To explore this question, we examined 436 individuals from the Alzheimer’s Disease Neuroimaging Initiative (ADNI) study using data collected from June 2010 to December 2021 and pulled on January 18, 2022. ADNI is a world-wide observational study launched in 2003 as a public-private partnership, led by principal investigator Michael W. Weiner, MD, and includes the extensions ADNI-GO (launched 2009), ADNI-2 (2011), and ADNI-3 (2016). The primary goal of ADNI has been to test whether serial magnetic resonance imaging (MRI), positron emission tomography (PET), other biological markers, and clinical and neuropsychological assessment can be combined to measure the progression of MCI and early AD. The study population is focused on older adults across the spectrum of AD, and individuals are followed up at approximately yearly intervals. Written informed consent was obtained by all ADNI participants at study entry, and the study was approved by each site’s respective institutional review board. Details and up-to-date information appear on the ADNI website.^20^ This manuscript conforms to the Strengthening the Reporting of Observational Studies in Epidemiology (STROBE) reporting guidelines for cohort studies.

The selection criteria for this study required both a participant and study partner response on the memory component of the Everyday Cognition (ECog) questionnaire from the same time point, a Clinical Dementia Rating (CDR) examination with global CDR score of 0 occurring within a year of their first ECog exam, and at least 2 follow-up CDR time points. Of note, for all except 3 individuals, their first ECog examination (hereafter, baseline) coincided with their first ADNI study visit. To further characterize the sample, demographic data on race and ethnicity were self-identified by the participant within investigator-defined categories (race: American Indian or Alaska Native, Asian, Black or African American, Native Hawaiian or other Pacific Islander, >1 race, White, and unknown; ethnicity: Hispanic or Latino, not Hispanic or Latino, and unknown) at study entry.

### Variables

#### Clinical Progression

Clinical progression was determined using the global score on the CDR. The participants in this study were all selected to have global CDR score of 0 at their baseline ECog exam. Clinical progression was then defined as the first occurrence of 2 consecutive time points with global CDR score of 0.5 or greater. Overall, 91 participants (20.9%) had progressed according to this definition when these data were pulled from the Laboratory of Neuro Imaging (LONI) database on January 18, 2022.

#### Memory Awareness

Memory awareness was assessed using the memory subscale of the ECog Questionnaire. There are 39 questions on the ECog, 8 of which are on the memory subscale. The participant and their study partner each separately rate the participant’s change compared with 10 years ago in ability to perform certain everyday tasks, such as remembering a few items without a shopping list or recalling conversations a few days later. Responses are on a Likert scale, with 1 indicating better or no change; 2, questionable or occasionally worse; 3, consistently a little worse; and 4, consistently much worse. Each question can instead be responded to with I do not know, which is treated as a missing value. Missing values across the sample for each item on the ECog questionnaire ranged from 0.13% to 2.78% and were handled with multiple imputation.

#### Traditional Awareness Score

The traditional memory awareness score was computed by subtracting the study partner’s response on each item from the participant’s response, then taking the mean of all item-level differences. Equivalently, the difference of the study partner’s mean response and participant’s mean response can be taken instead. Positive values indicate heightened awareness, and negative values indicate unawareness.

#### New Awareness Subscores

An unawareness subscore was computed by subtracting the study partner’s score from the participant’s score for each item, capping each positive item difference at zero (in effect treating instances of heightened awareness as the absence of unawareness), and taking the mean. A heightened awareness subscore was computed in an equivalent manner by capping each negative item difference at zero and taking the mean. Note that the new unawareness and heightened awareness subscores sum to the traditional awareness score.

### Statistical Analysis

All statistical analyses were performed in R version 4.2.0 (R Project for Statistical Computing). Multiple imputation was performed for missing ECog questionnaire items using the mice package (50 imputations; predictive mean matching).

Survival analyses were run using Cox regression models with the survival package to examine the association between each awareness measure at baseline and time to progression (measured in years from baseline). Outcome variables were progression status and (1) time to progression, for progressors, and (2) total time observed, for stable (ie, censored) individuals. One model was run for each of the 3 awareness measures, each adjusting for baseline age, sex, and years of education. Each model was run (1) with an outlying participant excluded, (2) including all participants, and (3) using robust standard errors.

Finally, longitudinal trajectories of each awareness measure were compared between individuals who progressed vs remained stable over their period of observation using linear mixed-effect models with the nlme package. Independent variables were age, sex, years of education, progression status (progressed vs stable), total period of observation for clinical progression (years), and the interaction of each with the time of the awareness measurement (years from baseline). Variance was modeled using correlated random slopes and intercepts. We additionally reran models removing the period of observation as an independent variable and instead imposing 3-, 6-, and 9-year cutoffs on the definition of progression, comparing those who progressed within 3, 6, or 9 years of observation with those who remained stable for at least 3, 6, or 9 years, respectively. Given the highly skewed, nonnormal distributions of the unawareness and heightened awareness subscores, we ran confirmatory quantile regression models using the lqmm package.

## Results

### Participants

**Figure 1.**
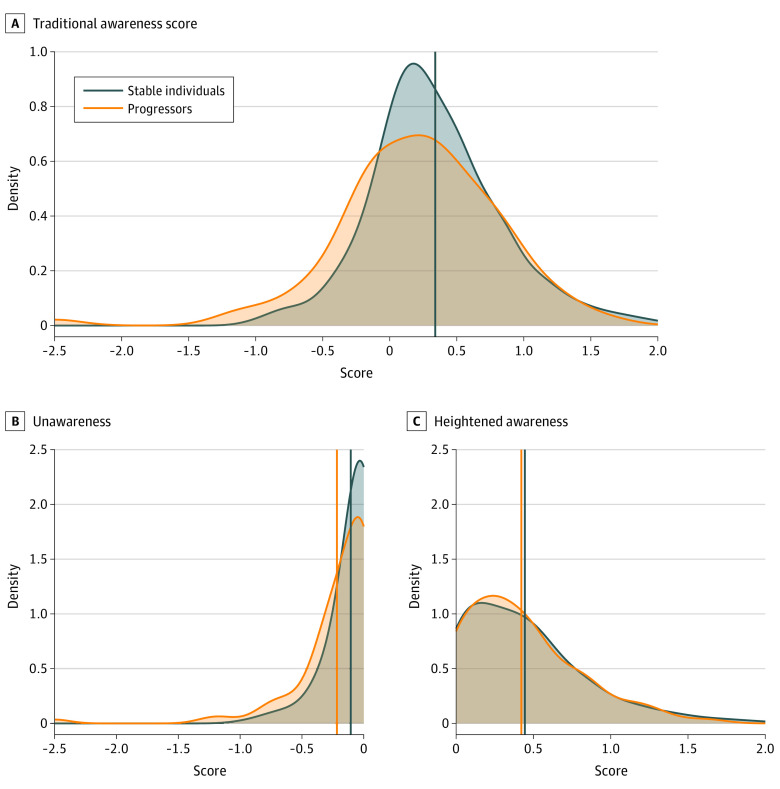
Distribution of Awareness Scores at Baseline by Progression Group Distribution plots of the (A) traditional awareness score, (B) unawareness subscore, and (C) heightened awareness subscore at baseline by progression group. Stable individuals are shown in blue, and progressors are shown in orange. Vertical lines indicate the means of each group.

The 436-person sample included 232 (53.2%) female participants, with a mean (SD) age of 74.5 (6.7) years and mean (SD) 16.43 (2.62) years of education. The sample included 25 (5.7%) Black participants, 14 (3.2%) Hispanic participants, and 398 (91.3%) White participants. The mean (SD) total length of CDR follow-up time was 5.48 (2.74) years, and 91 individuals (20.9%) clinically progressed over their period of observation. The period between consecutive CDR scores, as used to determine clinical progression, ranged from 0.5 to 3 years, with a mean (SD) period of 1.2 (0.6) years. The baseline mean (SD) of the traditional awareness score was 0.31 (0.51); for the unawareness subscore, it was −0.13 (0.23); and for the heightened awareness subscore, it was 0.44 (0.39). Distributions of these variables by progression group are shown in Figure 1. Missing values across the sample for each item on the ECog questionnaire ranged from 0.13% to 2.78% and were handled with multiple imputation. Participants’ baseline observation occurred between calendar years 2010 and 2019, with median year 2012. Demographic characteristics are detailed in Table 1.

**Table 1.** Baseline Sample Demographic Characteristics and Awareness Scores With Comparisons Between Progressors vs Stable Individuals

Characteristic	Participants, No. (%)			*P* value^a^
	Overall (N = 436)	Stable (n = 345)	Progressors (n = 91)	
Phase				
ADNI-GO	76 (17.4)	52 (15.1)	24 (26.4%)	.004
ADNI-2	299 (68.6)	237 (68.7)	62 (68.1%)	
ADNI-3	61 (14.0)	56 (16.2)	5 (5.5%)	
Age, mean (SD), y	74.5 (6.7)	74.1 (6.8)	76.1 (6.1)	.003
Education, mean (SD), y	16.43 (2.62)	16.56 (2.59)	15.95 (2.68)	.03
Sex				
Female	232 (53.2)	186 (53.9)	46 (50.5)	.57
Male	204 (46.8)	159 (46.1)	45 (49.5)	
Race				
American Indian or Alaskan Native	1 (0.2)	1 (0.3)	0	.92
Asian	5 (1.1)	5 (1.4)	0	
Black or African American	25 (5.7)	20 (5.8)	5 (5.5)	
More than 1 race	7 (1.6)	6 (1.7)	1 (1.1)	
White	398 (91.3)	313 (90.7)	85 (93.4)	
Ethnicity				
Hispanic or Latino	14 (3.2)	13 (3.8)	1 (1.1)	.39
Not Hispanic or Latino	419 (96.1)	329 (95.4)	90 (98.9)	
Unknown	3 (0.7)	3 (0.9)	0	
Total follow-up time, mean (SD), y	5.48 (2.74)	5.27 (2.75)	6.26 (2.59)	.002
Traditional awareness score, mean (SD)	0.31 (0.51)	0.34 (0.48)	0.20 (0.61)	.09
Unawareness subscore, mean (SD)	−0.13 (0.23)	−0.11 (0.18)	−0.22 (0.35)	<.001
Heightened awareness subscore, mean (SD)	0.44 (0.39)	0.44 (0.39)	0.42 (0.36)	.73

Abbreviation: ADNI, Alzheimer’s Disease Neuroimaging Initiative.

^a^ *P* values were calculated with Pearson χ^2^ test; Wilcoxon rank sum test; Fisher exact test.

The sample was arrived at as follows: out of an overall study enrollment of 1886 individuals across ADNI-GO, ADNI-2, and ADNI-3, a total of 1808 completed the memory portion of the ECog examination with a study partner. Of those, 1803 had a CDR examination within 1 year of their first ECog, 753 of those had a baseline global CDR score of 0, and 436 of those had at least 2 additional follow-up CDR time points—these individuals comprised the final sample. Demographic characteristics are compared between the final sample and individuals excluded for insufficient CDR follow-up in the eTable in Supplement 1.

### Survival Analysis

Three survival models were run, one for each awareness measure, to examine the association between time to progression and baseline awareness scores. One progressing individual with an outlying low traditional and unawareness score was excluded, and all models were confirmed to meet the proportional hazards assumption.

The traditional awareness score was not associated with progression, with a hazard ratio (HR) of 0.65 (95% CI, 0.41 to 1.02) for a 1-unit increase in score (*P* = .06). Equivalently interpreted, a 1-unit decrease would indicate a 55% increase in progression hazard (95% CI, −2.5% to 146%). The unawareness score was associated with progression, with a 1-unit increase in score associated with an 84% reduction in progression hazard (HR, 0.16 [95% CI, 0.07 to 0.35], *P* < .001). Equivalently interpreted, a 1-unit decrease in score was associated with a 540% increase in progression hazard (95% CI, 183% to 1347%). The heightened awareness score was not associated with progression (HR, 0.93 [95% CI, 0.53 to 1.62]; *P* = .80). Model results are presented in detail in Table 2.

**Table 2.** Results of the 3 Survival Analyses^a^

Variable	*b* (95% CI)^b^	HR (95% CI)	*t*	*P* value
**Traditional awareness model**				
Age	0.03 (0.00 to 0.06)	1.03 (1.00 to 1.07)	1.98	.05
Male sex	0.13 (−0.31 to 0.57)	1.14 (0.73 to 1.76)	0.59	.56
Years of education	−0.08 (−0.16 to 0.00)	0.92 (0.85 to 1.00)	−2.11	.04
Traditional awareness score	−0.44 (−0.90 to 0.02)	0.65 (0.41 to 1.02)	−1.89	.06
**Unawareness model**				
Age	0.03 (0.00 to 0.06)	1.03 (1.00 to 1.07)	2.00	.049
Male sex	0.09 (−0.35 to 0.52)	1.09 (0.70 to 1.69)	0.39	.70
Years of education	−0.08 (−0.15 to 0.00)	0.93 (0.86 to 1.00)	−1.97	.05
Unawareness subscore	−1.86 (−2.67 to −1.04)	0.16 (0.07 to 0.35)	−4.53	<.001
**Heightened awareness model**				
Age	0.03 (0.00 to 0.06)	1.03 (1.00 to 1.07)	2.03	.04
Male sex	0.17 (−0.27 to 0.60)	1.18 (0.76 to 1.82)	0.76	.45
Years of education	−0.08 (−0.16 to 0.00)	0.92 (0.86 to 1.00)	−2.02	.046
Heightened awareness subscore	−0.07 (−0.63 to 0.48)	0.93 (0.53 to 1.62)	−0.26	.80

Abbreviation: HR, hazard ratio.

^a^ Each model was run with a separate awareness score relating to the hazard of clinical progression. Models were adjusted for age, sex, and years of education. One individual with outlying traditional awareness and unawareness scores was excluded from those analyses.

^b^ *b* = log(HR) = unstandardized effect size.

When these analyses were rerun including the individual possessing outlying awareness scores, the traditional awareness model failed the proportional hazards assumption, and the association of that score with progression reached statistical significance (HR, 0.56 [95% CI, 0.36-0.89]; *P* = .02). All other results remained unchanged. Confirmatory robust Cox regression models produced equivalent results to those with the outlier excluded.

### Awareness Measures over Time

**Figure 2.**
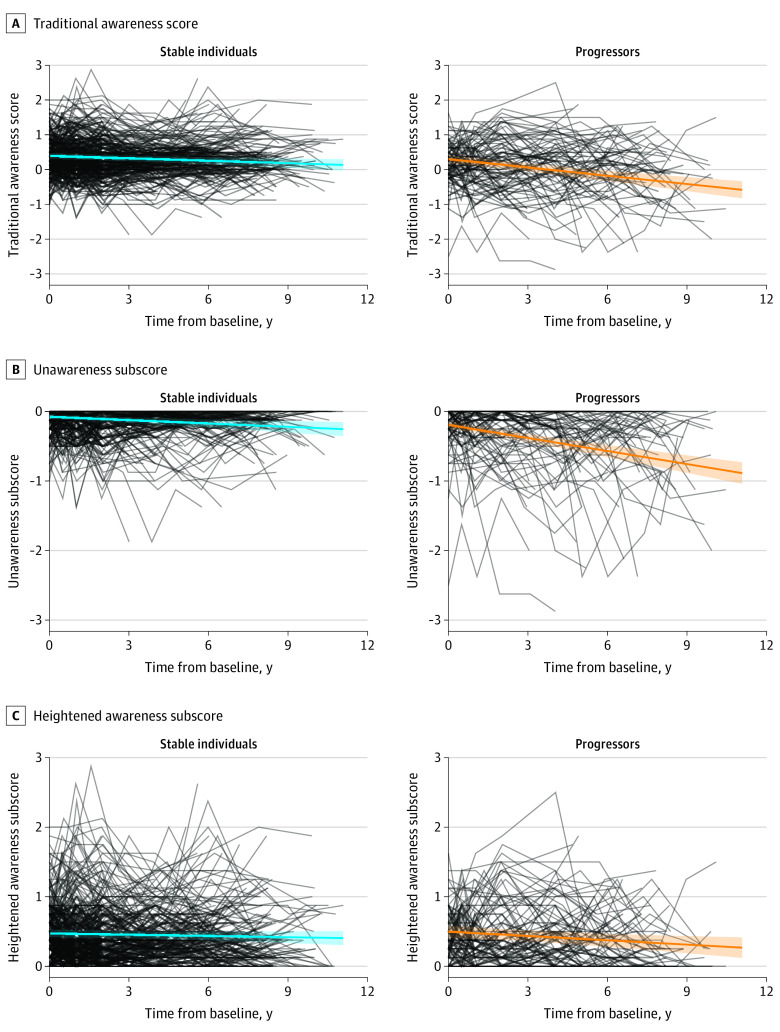
Awareness Scores Over Time by Progression Group With Linear Fit Spaghetti plots of (A) traditional awareness score, (B) unawareness subscore, and (C) heightened awareness subscore over time with linear fit by progression group. Black lines indicate individual observations over time, while blue (stable) and orange (progressors) lines indicate linear model fit, with shaded areas indicating 95% CIs.

The trajectory of each awareness score over time was compared between progressors and those who remained stable over their observed time frame. Each model showed a significant interaction between progression group and time, with progressors showing a steeper decline on every awareness measure (traditional awareness score: unstandardized *b* = −0.06 [95% CI, −0.08 to −0.03]; *t* = −4.74; *P* < .001; unawareness subscore: *b* = −0.05 [95% CI, −0.06 to −0.03]; *t* = −6.26; *P* < .001; heightened awareness subscore: *b* = −0.01 [95% CI, −0.03 to 0.00]; *t* = −2.02; *P* = .04). Models are presented in Table 3 and visualized in Figure 2. Confirmatory quantile regression models showed no change in results.

**Table 3.** Association Between Progression and Each Awareness Score Over Time for Various Progression Time Frame Cutoffs^a^

Model outcome variable	*b* (95% CI)^b^	*t*	*P* value
**Progression time frame: total time observed** ^c^			
Traditional awareness score	−0.06 (−0.08 to −0.03)	−4.74	<.001
Unawareness subscore	−0.05 (−0.06 to −0.03)	−6.26	<.001
Heightened awareness subscore	−0.01 (−0.03 to 0.00)	−2.02	.04
**Progression time frame: 3 y from baseline** ^d^			
Traditional awareness score	−0.04 (−0.07 to −0.01)	−2.34	.02
Unawareness subscore	−0.04 (−0.06 to −0.02)	−3.59	<.001
Heightened awareness subscore	−0.01 (−0.03 to 0.01)	−0.87	.39
**Progression time frame: 6 y from baseline** ^e^			
Traditional awareness score	−0.05 (−0.07 to −0.02)	−3.37	<.001
Unawareness subscore	−0.04 (−0.05 to −0.02)	−4.11	<.001
Heightened awareness subscore	−0.02 (−0.03 to 0.00)	−1.98	.048
**Progression time frame: 9 y from baseline** ^f^			
Traditional awareness score	−0.07 (−0.11 to −0.03)	−3.80	<.001
Unawareness subscore	−0.06 (−0.08 to −0.03)	−3.95	<.001
Heightened awareness subscore	−0.02 (−0.04 to 0.00)	−2.25	.02

^a^ Models were run with each awareness score as the outcome variable with baseline age, sex, years of education, progressing group, and the interaction of each with time from baseline as factors. In the model using the total time observed as the progression timeframe, each participant’s total time observed was additionally adjusted for. Each row presents statistics for the interaction term between time and progression group (progressed vs stable) for a separate model.

^b^ *b *is the unstandardized effect size.

^c^ A total of 436 participants (91 progressed, 345 remained stable over their total period of observation).

^d^ A total of 334 participants (57 progressed within 3 years; 278 remained stable for at least 3 years).

^e^ A total of 238 participants (79 progressed within 6 years; 159 remained stable for at least 6 years).

^f^ A total of 127 (91 progressed within 9 years; 36 remained stable for at least 9 years).

To better understand these patterns, we examined progression within a series of limited time frames, comparing individuals who progressed within 3, 6, or 9 years with those who remained stable for at least 3, 6, or 9 years, respectively. The results largely agreed with the previous analyses, showing a greater association between progression and the traditional and unawareness scores than with the heightened awareness score (Table 3). However, these models were each limited in their sample size, and confirmatory quantile regression models did not find the same significant difference as the linear mixed-effects models by 3- or 6-year progression group in the longitudinal trajectories of the traditional or unawareness scores.

## Discussion

In this study, we introduced 2 new awareness subscores—a heightened awareness score and an unawareness score—for the measurement of awareness of memory decline assessed using a participant and study partner administered questionnaire. When these new subscores were compared with each other and with the traditional awareness score, only the unawareness subscore was associated with future clinical progression for individuals who were CN at baseline. No association was found between future clinical progression and the traditional awareness score nor heightened awareness subscore at baseline. These results are in line with previous studies, which have shown an association between clinical progression and greater unawareness but not with heightened awareness.^6^ These new subscores present more direct, isolated measures of the 2 dimensions of unawareness than the traditional score, allowing for greater sensitivity and specificity in future investigations of the association between heightened awareness, unawareness, and AD.

Our longitudinal analysis of the trajectories of each of the 3 scores showed that progressors declined significantly more over time than stable individuals on all scores, with the unawareness subscore having the greatest effect size. These findings are in line with previous studies that have demonstrated longitudinal decline in awareness in CN participants who later progress to AD dementia,^8^ in amyloid-β–positive individuals vs amyloid-β–negative individuals,^6^ as well as in carriers vs noncarriers of the presenilin-1 alteration causing autosomal dominant AD.^7^ Additionally, greater baseline unawareness in MCI has been shown to be associated with future progression to AD dementia^21^ and amyloid-β status.^22^ Because of the way the traditional awareness score is calculated, a decline in the traditional score represents a progressive loss of awareness. Accordingly, it is unsurprising that the unawareness subscore follows the same path as the traditional score. The steeper decline in the heightened awareness subscore among progressors compared with stable individuals additionally fits in with this pattern and especially makes sense, as this subscore is not independent from the unawareness subscore.

The 3 scores differed in their baseline association with future clinical progression. In our survival analysis, only the unawareness subscore was significantly associated with future progression, with greater unawareness associated with greater risk of future progression. These results are in line with former studies showing that loss of awareness is associated with future clinical progression.^21^ Additionally, these results are supported by previous results showing an association between loss of awareness and a buildup of AD biomarkers,^6,7,10,11,12,17^ suggesting that participants harboring AD pathology who are on the path toward progression may show greater loss of awareness. The fact that the heightened awareness subscore was not significant in the survival analysis suggests that participants expressing more concerns than their study partners may not alone be an informative indicator of future progression. Heightened awareness may be nonspecific to AD, experienced by people as a result of normal aging as well as by those who will eventually progress. In contrast, unawareness is more specific to AD-related changes, possibly reflecting a decline in the person’s self-judgment of their own cognitive abilities as pathology increases.

### Limitations

There are several limitations to our study and to the use of these new subscores. Each of the subscores have notable floor and ceiling effects due to the imposed floors and ceilings in their calculations, and so viewing either score individually cannot present the full picture of any single individual’s state of awareness. Additionally, these nonnormal subscores may therefore be statistically cumbersome when investigated as outcome variables, compared with the more normally distributed traditional awareness score. Transformations, generalized linear models, and nonparametric approaches may be necessary to analyze these subscores appropriately, potentially making it more difficult to interpret the statistical outcomes. Although these limits are not present for the traditional awareness score, its own limitations justify the introduction of these 2 new subscores. With regard to study design, our sample from the ADNI data set is overwhelmingly White, non-Hispanic, and highly educated. This sample is not representative of the population as a whole, and future studies should confirm whether these results hold within more racially, ethnically, and socioeconomically diverse samples. Notably, there were also significant demographic differences between the final sample and individuals excluded for insufficient follow-up. However, this may be largely attributable to differences in ADNI phases—88% of the excluded participants were from ADNI-3, which (1) is currently ongoing and has not been followed up for as long, (2) has had follow-up impacted by the COVID-19 pandemic, and (3) enacted a diversity initiative in 2020 to accelerate enrollment of minorities. Revisiting this cohort in the future may provide sufficient follow-up for inclusion. Within our final sample, there were also significant demographic differences between our sample of progressors and stable individuals. The individuals who eventually progressed were significantly older, less educated, and were followed up for a longer length of time on average than the stable individuals. For these reasons, survival analyses were adjusted for age and education and longitudinal analyses for total time observed. Additionally, although the progressing group may be considered pure progressors, the stable group consists of a mix of truly stable CN individuals along with potential future progressors; for these reasons, longitudinal analyses comparing these groups should be interpreted with caution. Furthermore, the current study focused only on investigating these measures as they are associated with clinical status. Thus, future studies should investigate the associations of these measures to AD pathology.

## Conclusions

Our study demonstrates the benefit in decomposing the traditional awareness score into new heightened awareness and unawareness subscores. By isolating these 2 dimensions of awareness, we were able to investigate the associations of each independently. We found that heightened awareness in CN individuals was not specific to preclinical AD, showing no association with future clinical progression. Conversely, unawareness was associated with future clinical progression, with its subscore demonstrating an even stronger association than that of the traditional awareness score. When combined, the effects of heightened awareness and unawareness may obscure one another. However, when isolated, researchers and clinicians can more easily pick up on subtle changes in either dimension. These new subscores hold potential for the early detection of AD and intervention in clinic, as well as greater specificity and sensitivity in research into the relationship between awareness and AD.

## Notes

Supplement 1. **eTable.** Baseline Demographic Differences Between Final Sample and Individuals Excluded Due to Insufficient CDR Follow-up With Which to Determine Clinical Progression

Supplement 2.
Nonauthor Collaborators

Supplement 3.
Data Sharing Statement

## References

[1] Dubois B. The emergence of a new conceptual framework for Alzheimer’s disease. J Alzheimers Dis. 2018;62(3):1059-1066. doi:10.3233/JAD-170536 29036825 PMC5870001

[2] Dubois B, Feldman HH, Jacova C, . Advancing research diagnostic criteria for Alzheimer’s disease: the IWG-2 criteria. Lancet Neurol. 2014;13(6):614-629. doi:10.1016/S1474-4422(14)70090-0 24849862

[3] Jack CR Jr, Bennett DA, Blennow K, ; Contributors. NIA-AA research framework: toward a biological definition of Alzheimer’s disease. Alzheimers Dement. 2018;14(4):535-562. doi:10.1016/j.jalz.2018.02.018 29653606 PMC5958625

[4] Sperling RA, Aisen PS, Beckett LA, . Toward defining the preclinical stages of Alzheimer’s disease: recommendations from the National Institute on Aging–Alzheimer’s Association workgroups on diagnostic guidelines for Alzheimer’s disease. Alzheimers Dement. 2011;7(3):280-292. doi:10.1016/j.jalz.2011.03.003 21514248 PMC3220946

[5] Starkstein SE. Anosognosia in Alzheimer’s disease: diagnosis, frequency, mechanism and clinical correlates. Cortex. 2014;61:64-73. doi:10.1016/j.cortex.2014.07.019 25481465

[6] Hanseeuw BJ, Scott MR, Sikkes SAM, ; Alzheimer’s Disease Neuroimaging Initiative. Evolution of anosognosia in Alzheimer’s disease and its relationship to amyloid. Ann Neurol. 2020;87(2):267-280. doi:10.1002/ana.25649 31750553 PMC6980336

[7] Vannini P, Hanseeuw BJ, Gatchel JR, . Trajectory of unawareness of memory decline in individuals with autosomal dominant Alzheimer disease. JAMA Netw Open. 2020;3(12):e2027472. doi:10.1001/jamanetworkopen.2020.27472 33263761 PMC7711319

[8] Wilson RS, Boyle PA, Yu L, . Temporal course and pathologic basis of unawareness of memory loss in dementia. Neurology. 2015;85(11):984-991. doi:10.1212/WNL.0000000000001935 26311746 PMC4567465

[9] Amariglio RE, Becker JA, Carmasin J, . Subjective cognitive complaints and amyloid burden in cognitively normal older individuals. Neuropsychologia. 2012;50(12):2880-2886. doi:10.1016/j.neuropsychologia.2012.08.011 22940426 PMC3473106

[10] Cacciamani F, Tandetnik C, Gagliardi G, ; INSIGHT-PreAD study group. Low cognitive awareness, but not complaint, is a good marker of preclinical Alzheimer’s disease. J Alzheimers Dis. 2017;59(2):753-762. doi:10.1016/j.jalz.2017.06.2051 28671134

[11] Gagliardi G, Houot M, Cacciamani F, Habert MO, Dubois B, Epelbaum S; for ADNI; for the INSIGHT-preAD study group. The meta-memory ratio: a new cohort-independent way to measure cognitive awareness in asymptomatic individuals at risk for Alzheimer’s disease. Alzheimers Res Ther. 2020;12(1):57. doi:10.1186/s13195-020-00626-1 32408882 PMC7222501

[12] Perrotin A, Mormino EC, Madison CM, Hayenga AO, Jagust WJ. Subjective cognition and amyloid deposition imaging: a Pittsburgh Compound B positron emission tomography study in normal elderly individuals. Arch Neurol. 2012;69(2):223-229. doi:10.1001/archneurol.2011.666 22332189 PMC4004919

[13] Visser PJ, Verhey F, Knol DL, . Prevalence and prognostic value of CSF markers of Alzheimer’s disease pathology in patients with subjective cognitive impairment or mild cognitive impairment in the DESCRIPA study: a prospective cohort study. Lancet Neurol. 2009;8(7):619-627. doi:10.1016/S1474-4422(09)70139-5 19523877

[14] Gagliardi G, Kuppe M, Lois C, Hanseeuw B, Vannini P; Alzheimer’s Disease Neuroimaging Initiative. Pathological correlates of impaired self-awareness of memory function in Alzheimer’s disease. Alzheimers Res Ther. 2021;13(1):118. doi:10.1186/s13195-021-00856-x 34172086 PMC8234669

[15] Dalla Barba G, La Corte V, Dubois B. For a cognitive model of subjective memory awareness. J Alzheimers Dis. 2015;48(s1)(suppl 1):S57-S61. doi:10.3233/JAD-150141 26402084

[16] Vannini P, Hanseeuw B, Munro CE, . Anosognosia for memory deficits in mild cognitive impairment: insight into the neural mechanism using functional and molecular imaging. Neuroimage Clin. 2017;15:408-414. doi:10.1016/j.nicl.2017.05.020 28616381 PMC5458095

[17] Cacciamani F, Sambati L, Houot M, Habert MO, Dubois B, Epelbaum S; INSIGHT-PreAD study group. Awareness of cognitive decline trajectories in asymptomatic individuals at risk for AD. Alzheimers Res Ther. 2020;12(1):129. doi:10.1186/s13195-020-00700-8 33054821 PMC7557018

[18] Clare L, Marková IS, Roth I, Morris RG. Awareness in Alzheimer’s disease and associated dementias: theoretical framework and clinical implications. Aging Ment Health. 2011;15(8):936-944. doi:10.1080/13607863.2011.583630 21702711

[19] Clare L, Marková I, Verhey F, Kenny G. Awareness in dementia: a review of assessment methods and measures. Aging Ment Health. 2005;9(5):394-413. doi:10.1080/13607860500142903 16024399

[20] ADNI. Accessed March 30, 2023. https://adni.loni.usc.edu/

[21] Munro CE, Donovan NJ, Amariglio RE, . The impact of awareness of and concern about memory performance on the prediction of progression from mild cognitive impairment to Alzheimer disease dementia. Am J Geriatr Psychiatry. 2018;26(8):896-904. doi:10.1016/j.jagp.2018.04.008 29866588 PMC6959130

[22] Sánchez-Benavides G, Salvadó G, Arenaza-Urquijo EM, ; ALFA Study. Quantitative informant- and self-reports of subjective cognitive decline predict amyloid beta PET outcomes in cognitively unimpaired individuals independently of age and *APOE ε4.* Alzheimers Dement (Amst). 2020;12(1):e12127. doi:10.1002/dad2.12127 33204815 PMC7656171

