# Supplementary material for: Measurement of Dimensions of Self-awareness of Memory Function and Their Association With Clinical Progression in Cognitively Normal Older Adults
Source: JAMA Netw Open. 2023 Apr 25;6(4):e239964. doi: 10.1001/jamanetworkopen.2023.9964 (PMC10130951; doi:10.1001/jamanetworkopen.2023.9964)
Supplement: Supplement 1. — eTable. Baseline Demographic Differences Between Final Sample and Individuals Excluded Due to Insufficient CDR Follow-up With Which to Determine Clinical Progression [file jamanetwopen-e239964-s001.pdf]

# Supplemental Online Content

Mimmack KJ, Gagliardi GP, Marshall GA, Vannini P; Alzheimer's Disease Neuroimaging Initiative. Measurement of dimensions of self-awareness of memory function and their association with clinical progression in cognitively normal older adults. *JAMA Netw Open.* 2023;6(4):e239964. doi:10.1001/jamanetworkopen.2023.9964

**eTable.** Baseline Demographic Differences Between Final Sample and Individuals Excluded Due to Insufficient CDR Follow-up With Which to Determine Clinical Progression

This supplemental material has been provided by the authors to give readers additional information about their work.

© 2023 Mimmack KJ et al. *JAMA Network Open.*

*eTable. Baseline Demographic Differences Between Final Sample and Individuals Excluded Due to Insufficient CDR Follow-up With Which to Determine Clinical Progression*

| **Characteristic** | **Overall (N = 753)**[a] | **Sample (N = 436)**[a] | **Excluded (N = 317)**[a] | ***p*-value**[b] |
|---|---|---|---|---|
| **Phase** | | | | <0.001*** |
| ADNIGO | 82 (11%) | 76 (17%) | 6 (1.9%) | |
| ADNI2 | 330 (44%) | 299 (69%) | 31 (9.8%) | |
| ADNI3 | 341 (45%) | 61 (14%) | 280 (88%) | |
| **Age** | 72.9 (7.1) | 74.5 (6.7) | 70.7 (7.0) | <0.001*** |
| **Education, Years** | 16.57 (2.47) | 16.43 (2.62) | 16.75 (2.24) | 0.17 |
| **Gender** | | | | 0.02* |
| F | 427 (57%) | 232 (53%) | 195 (62%) | |
| M | 326 (43%) | 204 (47%) | 122 (38%) | |
| **Race** | | | | <0.001*** |
| American Indian or Alaskan Native | 2 (0.3%) | 1 (0.2%) | 1 (0.3%) | |
| Asian | 23 (3.1%) | 5 (1.1%) | 18 (5.7%) | |
| Black or African American | 68 (9.0%) | 25 (5.7%) | 43 (14%) | |
| More than one race | 12 (1.6%) | 7 (1.6%) | 5 (1.6%) | |
| White | 644 (86%) | 398 (91%) | 246 (78%) | |
| Unknown | 4 (0.5%) | 0 (0%) | 4 (1.3%) | |
| **Ethnicity** | | | | <0.001*** |
| Hispanic or Latino | 47 (6.2%) | 14 (3.2%) | 33 (10%) | |
| Not Hispanic or Latino | 703 (93%) | 419 (96%) | 284 (90%) | |
| Unknown | 3 (0.4%) | 3 (0.7%) | 0 (0%) | |
| **Total Follow-Up Time, Years** | 3.62 (3.11) | 5.48 (2.74) | 1.06 (1.15) | <0.001*** |
| **Traditional Awareness Score** | 0.31 (0.52) | 0.31 (0.51) | 0.31 (0.52) | 0.55 |
| **Unawareness Sub-Score** | -0.13 (0.22) | -0.13 (0.23) | -0.12 (0.21) | 0.72 |
| **Heightened Awareness Sub-Score** | 0.43 (0.40) | 0.44 (0.38) | 0.43 (0.41) | 0.35 |

[a] n (%); Mean (SD)

[b] Pearson's Chi-squared test; Wilcoxon rank sum test; Fisher's exact test

© 2023 Mimmack KJ et al. *JAMA Network Open.*
