# Supplementary material for: Measurement of Dimensions of Self-awareness of Memory Function and Their Association With Clinical Progression in Cognitively Normal Older Adults
Source: JAMA Netw Open. 2023 Apr 25;6(4):e239964. doi: 10.1001/jamanetworkopen.2023.9964 (PMC10130951; doi:10.1001/jamanetworkopen.2023.9964)
Supplement: Supplement 2. — Nonauthor Collaborators [file jamanetwopen-e239964-s002.pdf]

Supplemental Online Content: Nonauthor Collaborators
*First name, last name, and suffix (if applicable) are required and will appear in PubMed.

*Group Name(s): Alzheimer's Disease Neuroimaging Initiative							
***First Name and Middle Initial(s)**	***Last Name**	***Suffix (eg, Jr, III)**	**Academic Degrees**	**Institution**	**Location (city, state/province, country)**	**Role or Contribution, eg, chair, principal investigator**	**Group (if more than 1 Group listed in the byline) and/or Subgroup (eg, Steering Committee)**
Michael W.	Weiner		M.D.	UCSF, NCIRE, VA Medical Center	San Francisco, CA, USA	Principal Investigator	Alzheimer's Disease Neuroimaging Initiative (ADNI): Administrative Core
Arthur W.	Toga		Ph.D.	Laboratory of Neuro Imaging (LONI); University of Southern California	Los Angeles, CA, USA	Principal Investigator	Alzheimer's Disease Neuroimaging Initiative (ADNI): Informatics Core
Laurel	Beckett		Ph.D.	University of California, Davis	Davis, CA, USA	Principal Investigator	Alzheimer's Disease Neuroimaging Initiative (ADNI): Biostatistics Core
Paul	Aisen		M.D.	University of Southern California	Los Angeles, CA, USA	Principal Investigator	Alzheimer's Disease Neuroimaging Initiative (ADNI): Clinical Core / Coordinating Center
Ronald	Petersen		M.D., Ph.D.	Mayo Clinic	Rochester, MN, USA	Principal Investigator	Alzheimer's Disease Neuroimaging Initiative (ADNI): Clinical Core / Coordinating Center
Joseph	Locascio		Ph.D.	Harvard Catalyst; Massachusetts General Hospital	Boston, MA, USA	Statistical Consultant	N/A (referenced in acknowledgements)
