# Supplementary material for: Measurement of Dimensions of Self-awareness of Memory Function and Their Association With Clinical Progression in Cognitively Normal Older Adults
Source: JAMA Netw Open. 2023 Apr 25;6(4):e239964. doi: 10.1001/jamanetworkopen.2023.9964 (PMC10130951; doi:10.1001/jamanetworkopen.2023.9964)
Supplement: Supplement 3. — Data Sharing Statement [file jamanetwopen-e239964-s003.pdf]

Data Sharing Statement

Mimmack. Measurement of Dimensions of Self-awareness of Memory Function and Their Association With Clinical Progression in Cognitively Normal Older Adults. *JAMA Netw Open*. Published April 25, 2023. doi:10.1001/jamanetworkopen.2023.9964

Data
Data available: No

Additional Information
Explanation for why data not available: Data for this study are from ADNI and thus is available to the public to download.
